# Development and Validation of an Algorithm for Foot Contact Detection in High-Dynamic Sports Movements Using Inertial Measurement Units

**DOI:** 10.3390/s26030988

**Published:** 2026-02-03

**Authors:** Stefano Di Paolo, Margherita Mendicino, José Miguel Palha de Araújo dos Santos, Eline Nijmeijer, Pieter Heuvelmans, Francesco Della Villa, Alli Gokeler, Anne Benjaminse, Stefano Zaffagnini

**Affiliations:** 1 2nd Orthopaedic and Traumatological Clinic, IRCCS Rizzoli Orthopaedic Institute, 40136 Bologna, Italy; stefano.dipaolo@ior.it (S.D.P.); stefano.zaffagnini@unibo.it (S.Z.); 2 Faculdade de Engenharia da Universidade do Porto (FEUP), University of Porto, 4200-465 Porto, Portugal; up202007566@edu.fe.up.pt; 3 Department of Human Movement Sciences, University Medical Center Groningen, University of Groningen, 9713 AV Groningen, The Netherlands; e.m.nijmeijer@umcg.nl (E.N.); a.benjaminse@umcg.nl (A.B.); 4 Exercise Science and Neuroscience Unit, Department of Exercise and Health, Paderborn University, 33098 Paderborn, Germany; pieter.heuvelmans@uni-paderborn.de (P.H.); alli.gokeler@uni-paderborn.de (A.G.); 5 Education and Research Department, Isokinetic Medical Group, FIFA Medical Centre of Excellence, 40132 Bologna, Italy; f.dellavilla@isokinetic.com; 6 Faculty of Health, Master Performance Sport and Health, Amsterdam University of Applied Sciences, 1105 BD Amsterdam, The Netherlands; 7 Department of Biomedical and Neuromotor Sciences, University of Bologna, 40126 Bologna, Italy

**Keywords:** footstep detection, wearables, algorithm validation, change of direction, ACL injury, biomechanics

## Abstract

**Highlights:**

**What are the main findings?**

- A novel IMU-based algorithm combining pelvis vertical velocity (PVV) and the resultant foot acceleration (RFA) signals accurately detected events of initial contact and toe-off during high-dynamic sports movements.
- The algorithm outperforms individual PVV or RFA methods, achieving median offsets below 20 ms compared to force platform data.

**What is the implication of the main finding?**

- The algorithm enables reliable, field-based biomechanical analysis of complex movements, providing a portable and practical alternative to lab-based instrumentation.
- The algorithm can support performance monitoring, injury risk assessment, and rehabilitation in real-world sport settings through IMUs, enhancing ecological validity and accessibility for athletes and clinicians.

**Abstract:**

Precise foot contact detection (FCD) is essential for accurate biomechanical analysis in sport performance, injury prevention, and rehabilitation. This study developed and validated an inertial measurement units (IMUs)-based algorithm for FCD during sports movements. Thirty-four healthy athletes (22.8 ± 4.1 years old) performed 90° changes of direction and sprints with deceleration. Data were collected via a force platform (AMTI, 1000 Hz) and a full-body IMU suit (MTw Awinda, Movella, 60 Hz). Two IMU-based algorithms relying on pelvis vertical velocity (PVV) and resultant foot acceleration (RFA), respectively, were tested to detect initial contact (IC) and toe-off (TO). Force platform data served as the gold standard for comparison. Agreement was quantified through median offset and interquartile range (IQR); the influence of task, sex, leg, speed, and acceleration was investigated. The PVV algorithm showed higher offset than RFA for IC detection (16.7 ms vs. 10.2 ms) with comparable IQR and a substantially higher offset for TO (102.8 ms vs. 20.4 ms). Minimal influence of co-factors emerged (variance < 10%). Results were sensibly improved by combining PVV and RFA, for both IC (5.6 [70.4] ms) and TO (20.4 [78.7] ms). This algorithm offers a robust, portable alternative to force platforms, enabling accurate footstep detection and analysis of complex, sports movements in real-world environments, enhancing the ecological validity of sport assessments.

## 1. Introduction

Precise foot contact detection (FCD) is essential in biomechanical analysis, providing accurate information for optimizing sports performance, preventing injuries, and supporting rehabilitation. In sports movement analysis, identifying key events within the foot contact window is crucial for extracting temporal parameters, estimating ground reaction forces, and detecting asymmetries or compensatory strategies. This is especially important in multidirectional sports such as football, handball, and basketball, where accurately determining the stance phase is central to assessing musculoskeletal injury risk, including anterior cruciate ligament (ACL) rupture [1,2,3,4,5].

Traditionally, the detection of foot contact events relies on laboratory-based force platforms or optical motion capture systems, which restrict testing to controlled environments and to limited movement tasks, thus reducing the ecological validity of data collection by failing to reproduce the environmental complexity of high-demand sports situations. As research and its application in sports moves toward on-field assessment, wearable inertial measurement units (IMUs) have emerged as a viable alternative to laboratory-based motion capture in biomechanical studies, providing continuous monitoring in ecological and sport-specific conditions such as training and competitive settings [6,7,8].

A reliable method for proper FCD directly from IMU signals is required to ensure accurate IMU-based kinematics when force platform data are not available [6]. This has led to a proliferation of automatic FCD algorithms, which, however, have mostly been explored in the context of walking or straight-line running [9,10,11]. Indeed, their applicability and reliability for high-dynamic sports movements, such as cutting maneuvers, remain poorly understood.

Previous research underlined poor accuracy in applying gait-based FCD algorithms to high-dynamics, multidirectional movements [12]. These limitations particularly arise because gait-based algorithms assume regular steps and low accelerations, which are violated during rapid cutting maneuvers [13]. For instance, Falbriard et al. [14] showed that even during straight running, speed and foot-strike variations can cause errors up to 15 ms, suggesting larger errors in more complex movements. This is supported by Fadillioglu et al. [15], who reported task-dependent gait event detection accuracy, with directional changes leading to higher errors. Similarly, Niswander & Kontson [12] demonstrated that algorithm accuracy varies across walking tasks, such as obstacle navigation, highlighting how algorithm performance strongly depends on the movement context.

In addition, a recently published systematic review [16] synthesized existing IMU-based methods for FCD, highlighting the limited availability of algorithms for sports applications, with 92% of included studies investigating algorithms for linear running and sprinting tasks only. The sensitivity of existing IMU-based FCD algorithms to variations in movement speed has also been questioned [16,17,18], further evidencing the lack of robustness under realistic sports conditions.

Despite the growing adoption of wearable sensors for biomechanics and performance assessment in real-world settings [7,19], these recent findings highlight a significant gap in the literature, which limits their practical applicability due to the lack of validated algorithms for high-dynamic sports movements. Therefore, a broader adoption of IMUs in field-based biomechanical analyses, for both performance and clinical studies, also relies on the challenge of FCD in such movements.

Building on previous findings and addressing the gaps identified in the literature, the present study aimed to develop and validate an IMU-based FCD algorithm specifically designed for high-dynamic sports movements. Specifically, the concurrent validity of two literature-based algorithms was first analyzed against the ground truth. Based on their performance, a third hybrid algorithm was developed by integrating complementary features from the first two methods and validated against the gold standard.

## 2. Materials and Methods

### 2.1. Participants

Thirty-four competitive young, healthy athletes (22.8 ± 4.1 years, 18 males and 16 females, Tegner Level 9) were involved in this study. The inclusion criteria were an age between 18 and 50 years, a Tegner level of at least 5, and a BMI < 35 at the time of data collection. The exclusion criteria included a history of major musculoskeletal injuries or lower-limb surgery, the presence of cardiopulmonary or cardiovascular disorders, and any medical condition potentially limiting the safe execution of high-intensity movements [8,20].

Each athlete signed an informed consent form before entering this study. The research study was approved by the Institutional Review Board (IRB approval: 555/2018/Sper/IOR of 12 September 2018) of Area Vasta Emilia Romagna Centro (AVEC, Bologna, Italy) and registered on ClinicalTrials.gov (Identifier: NCT03840551).

### 2.2. Data Collection

**Figure 1:**
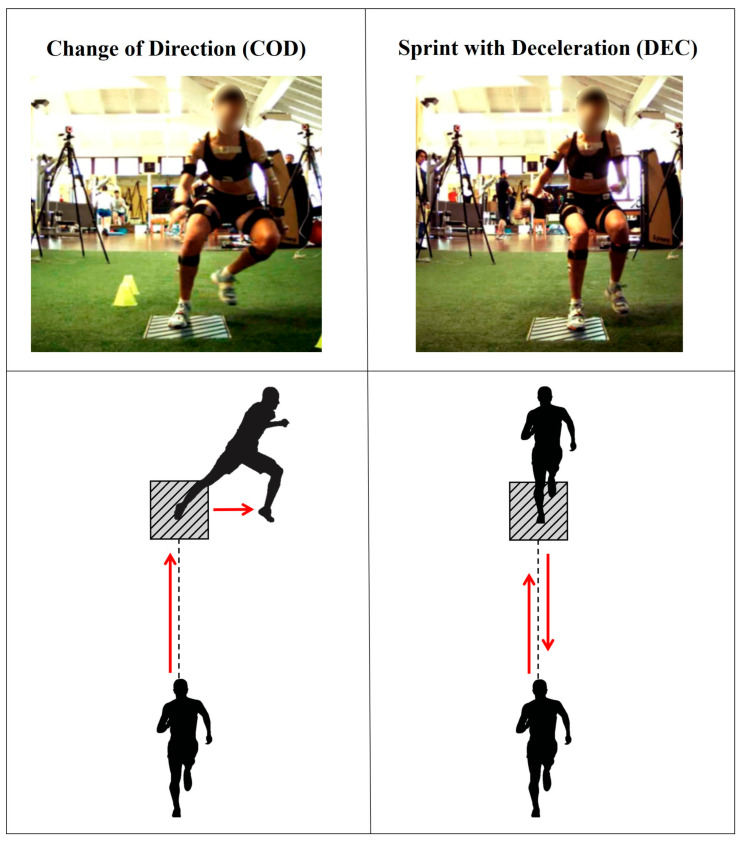
Example of a player equipped with IMUs, performing a sidestep change of direction (**left**) and a sprint with deceleration (**right**). In both representations, the player’s left foot makes contact with the force platform included in the setup. The schematic representations (bottom) of the tasks show the direction of the movement (red arrows) and the force platform (grey box).

Data collection was conducted at the Education and Research Department of the Isokinetic Medical Group in Bologna (Italy), in a laboratory equipped with artificial turf [19]. After a dynamic warm-up, athletes performed two high-dynamic tasks at maximum effort: a pre-planned 90° change of direction (COD) and a linear sprint with deceleration (DEC). The COD task consisted of a 5 m linear sprint, followed by a 90° sidestep cut and a further 3 m frontal sprint in the new direction, whereas the DEC task involved a 5 m sprint followed by a single-leg stop and a backward sprint while facing the same direction (Figure 1), as described in detail by Della Villa et al. (2022) [21]. These movements mimic common pivoting sports situations, such as pressing and cutting, widely recognized as situational patterns associated with ACL rupture [5,22,23,24,25]. Two to three valid trials (adequate effort, full foot contact on the platform) per side were recorded for each task. A sports medicine physician specializing in sports biomechanics (F.D.V.) supervised the athletes, provided standardized instructions to perform each task at their best, and verified the validity of the trials.

Ground truth force data were collected using a force platform embedded in the floor (AMTI 400*600, Watertown, MA, USA; 1000 Hz). Additionally, a set of 15 wearable IMUs (MTw Awinda, Movella, Enschede, The Netherlands; 60 Hz), placed by a single experienced operator (S.D.P.) on the upper limbs, trunk, and lower limbs, was used to collect full-body joint kinematics. Specifically, IMUs were positioned bilaterally on the dorsum of the feet, shanks, thighs, upper arms, forearms, and shoulders, as well as on the pelvis, sternum, and head. All sensors were secured using elastic straps and medical-grade adhesive tape, following the manufacturer’s instructions, to minimize displacement and ensure stable attachment throughout the movements. The system calibration was performed in both static (upright standing) and dynamic (walking) conditions. The wearable system was validated in the assessment of high-dynamic movements in previous studies [26,27,28]. IMUs and the force platform were synchronized via a hardware trigger during data acquisition for direct time comparison. Synchronization was verified by testing the trigger alignment in the first trials and periodically re-testing it during data collection. In addition, visual verification was performed on all trials after data collection by inspecting the temporal alignment of the signals. Trials showing evident temporal shifts indicative of synchronization errors were excluded from further analysis.

### 2.3. Data Processing

The data processing was performed in the proprietary IMU software (MVN Analyze 2023.2) to export full-body kinematics. Further analyses were performed in a custom MATLAB script (MATLAB R2023b, The MathWorks, Natick, MA, USA), using the Biomechanical ToolKit (BTK, v0.3.0), the Signal Processing Toolbox, and the Statistics and Machine Learning Toolbox.

A reference foot-contact window was defined using the vertical ground reaction force (vGRF) signal to detect initial contact (IC) and toe-off (TO) for each trial. IC was determined as the first frame in which the vGRF exceeded a 50 N threshold, and TO as the first frame in which the vGRF dropped below the same threshold [29,30]. The vGRF signal was then down-sampled to the IMU sampling frequency using the “resample” MATLAB function. The timing of IC and TO events was then resampled accordingly to ensure temporal alignment with the IMU data for proper comparison.

**Figure 2:**
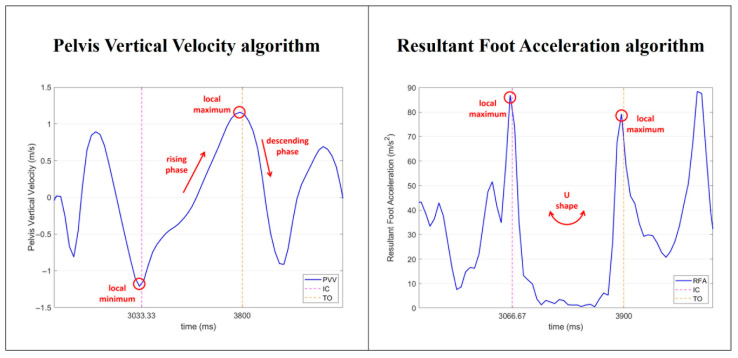
Example of pelvis vertical velocity curve (PVV, **left**) and resultant foot acceleration curve (RFA, **right**). Red annotations highlight key phases for recognizing the patterns for FCD. In PVV, the algorithm detects initial contact (pink dashed line) at the local minimum and toe-off (orange dashed line) at the following local maximum. The local minimum and subsequent local maximum define a rising phase that is followed by a drop in the curve, after toe-off. In RFA, the algorithm detects initial contact (pink dashed line) and toe-off (orange dashed line) at local maxima. A “U shape” phase is recognized in between the two points.

Two different algorithms were developed to identify IC and TO events for accurate FCD from IMU data. These rely on commonly used IMU signals in the literature for FCD in running or sprinting tasks [16]. Specifically, the first algorithm relied on pelvis vertical velocity (PVV, 1), retrieved from the IMU sensor output at the pelvis [29], while the second relied on resultant foot acceleration (RFA, 2), computed as the magnitude of the three-axis linear acceleration measured by the foot-mounted IMU [14,31,32,33]. A detailed description of the algorithms is provided in the subsubsections below, and graphical overview of these algorithms is shown in Figure 2.

#### 2.3.1. Algorithm 1: Pelvis Vertical Velocity (PVV)

The PVV algorithm detects IC and TO events based on the vertical component (*z*-axis) of the pelvis velocity, directly retrieved from the IMU sensor output at the pelvis [29]. The aim of the algorithm is to recognize a pattern in the PVV signal (Figure 2). First, the PVV signal is filtered by applying a low-pass third-order Butterworth filter (20 Hz cutoff frequency) to minimize the influence of IMU fixation artifacts [30]. Then, the algorithm identifies all local minima in the PVV signal as potential ICs of the FC window of interest and all local maxima as potential TOs. For each potential IC, the algorithm examines all possible TOs and checks three conditions: (1) TO occurs after the IC; (2) a descent in velocity occurs after the TO, meaning that the derivative of velocity must drop below a threshold set at −0.1 m/s^2^ (empirically chosen); and (3) the TO is the highest of peaks before the descent. If all these conditions are met, the IC and TO are selected. Otherwise, the algorithm moves on to the next potential TO.

#### 2.3.2. Algorithm 2: Resultant Foot Acceleration (RFA)

The RFA algorithm uses the resultant foot acceleration, computed as the magnitude of the three-axis linear acceleration measured by the foot-mounted IMU, to detect IC and TO events [14,31,32,33]. As for the PVV algorithm, the aim is to recognize a pattern in the RFA signal (Figure 2). First, the algorithm extracts the foot acceleration data along the x, y, and z axes and computes the resultant acceleration. The RFA signal is filtered with a low-pass third-order Butterworth filter (20 Hz cutoff frequency), to smooth the signal and remove high-frequency noise [14,32]. Then, the algorithm identifies the IC and TO events by following a sequence of steps: it detects all local maxima in the RFA signal, marking them as potentially desired ICs and TOs. For each potential IC, the algorithm examines the following potential TOs to verify whether they are greater than or equal to a defined threshold for TO peak detection, which is set at 30 m/s^2^ (empirically chosen). This threshold represents the minimum RFA value required for an eligible TO. If a potential TO does not meet this criterion, it is discarded, and the algorithm continues evaluating the next potential TO event.

Finally, the two approaches were combined into a hybrid algorithm to assess whether this yielded more accurate results compared to the reference system. Both PVV and RFA algorithms were coded to potentially detect all foot contact events within each trial. For the purposes of this study, the step, or foot contact, with the longest duration was selected as the valid one corresponding to the COD or DEC event [34]. If no valid IC and TO were found, the algorithms report an error message.

### 2.4. Algorithms’ Validation and Statistical Analysis

The IC and TO events detected by the IMU-based FCD algorithms were compared with those obtained from force platform measurements to assess concurrent validity. The consistency of each algorithm against the reference system was first inspected through Bland–Altman analysis, including mean offset, limits of agreement, and number of outliers. Additionally, the median offset (difference between force platform and IMU events’ time) and interquartile range (IQR) were used to quantify the agreement.

To further evaluate the performance of the algorithms, a Linear Mixed-Effects model was applied, investigating whether algorithm error was influenced by the following confounding factors: task category (COD/DEC), sex (male/female), and leg (dominant/non-dominant), while accounting for repeated measures across participants by including participant identification number as a random effect. Additionally, Pearson’s correlation coefficient was computed to examine potential error sources at IC and TO, focusing on movement speed for the PVV algorithm and the magnitude of acceleration for RFA, as these directly influence the respective signal characteristics.

Lastly, a hybrid algorithm was implemented by integrating the PVV and RFA approaches to combine their individual strengths and minimize the offset from the ground truth. The hybrid algorithm, which builds on the results of the individual analyses, is described in the Section 3.2 entitled “Algorithm 3: Hybrid”.

## 3. Results

A total of 302 valid trials were collected (164 COD, 138 DEC). Forty-five trials were excluded due to errors in manual triggering, resulting in IMU misalignment with the reference system. Consequently, 257 trials were considered for analysis.

The average FC window identified by the reference system was 478.3 ± 162.1 [459.9; 496.6] ms.

### 3.1. PVV and RFA Algorithms’ Performance

The average FC window identified by the IMU-based algorithms was 415.6 ± 185.0 [394.6; 436.5] ms for PVV and 456.0 ± 175.5 [436.1; 475.9] ms for RFA.

The PVV algorithm showed a higher median offset than RFA for IC detection (16.7 ms vs. 10.2 ms) with comparable IQR and a substantially higher offset for TO (102.8 ms vs. 20.4 ms) (Table 1). Both algorithms failed to detect IC and TO in one specific file (1 trial out of 257), due to the absence of a recognizable pattern.

**Figure 3:**
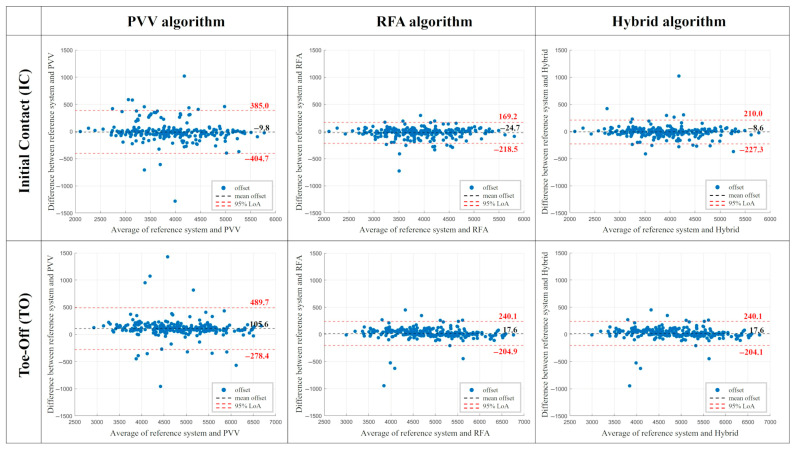
Bland–Altman plots for the three developed algorithms (PVV, RFA, hybrid) for both IC (**top row**) and TO detection (**bottom row**). Limits of agreement (red dashed lines) and mean offset (black dashed line) are reported. All values are in milliseconds (ms). Positive values indicate that the algorithm detected the foot contact event earlier than the reference system.

From the Bland–Altman analysis (Figure 3) and the median offsets, both the PVV and RFA algorithms showed a tendency to detect IC events later (negative offset) and TO events earlier (positive offset) than the reference system. Narrower limits of agreement for the RFA algorithm compared to the PVV were noted. RFA exhibited a higher mean offset for IC detection (−24.7 ms vs. −9.8 ms). The number of outliers ranged between 10 and 14 (less than 5.4% of trials) for both PVV and RFA algorithms, in both IC and TO detection.

The Linear Mixed-Effects model (Table 2) revealed that PVV offset was not statistically explained by any factor (*p* = 0.822 for IC; *p* = 0.465 for TO). In contrast, RFA offset (R^2^-adjusted = 0.071–0.072, *p* < 0.001) was explained by sex for IC detection (β = 3.242, *p* < 0.001), with male participants showing higher error and by task for TO detection (β = −3.785, *p* < 0.001), with a lower error observed in COD tasks. No between-subject variability was observed. Negative correlation was observed between acceleration magnitude and RFA error at IC (r = −0.33, *p* < 0.001), whereas no significant correlation was found at TO. No significant correlation was found between movement speed and PVV offset at both IC and TO (Appendix A).

The average resultant acceleration of the foot at the instant of IC, computed across all trials, was 94.6 ± 36.8 m/s^2^. In 45 out of 257 trials, the resultant foot acceleration at IC was below 57.8 m/s^2^, corresponding to one standard deviation below the mean across all trials. In these trials, PVV showed better performance than RFA (median offset of 53.7 ms vs. 77.8 ms) in detecting IC.

### 3.2. Algorithm 3: Hybrid

The average FC window identified by the hybrid IMU-based algorithms was 465.7 ± 180.7 [445.2; 486.2] ms.

**Figure 4:**
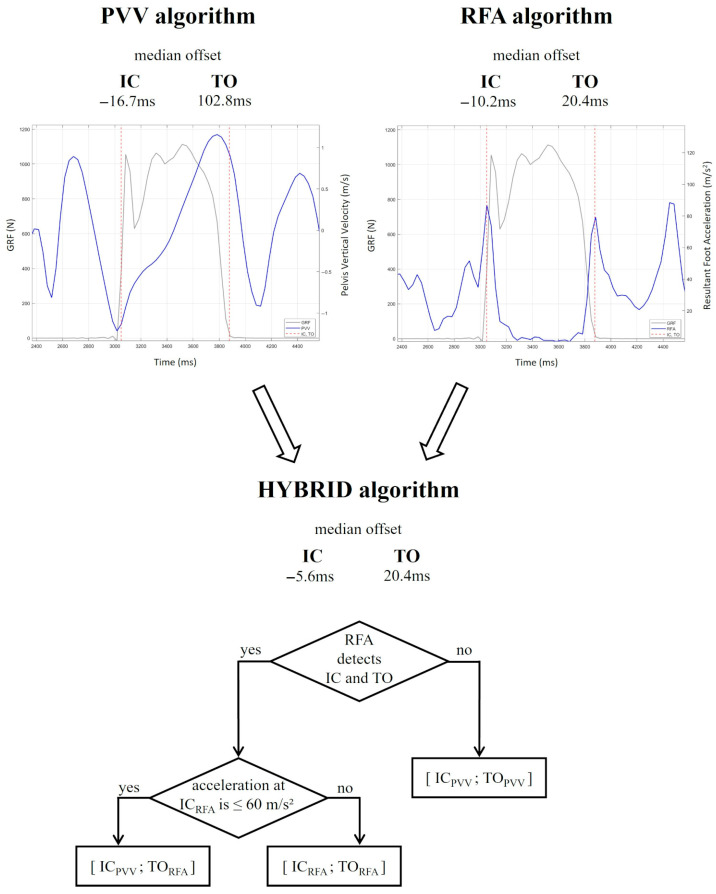
Methodological workflow diagram for hybrid foot contact detection algorithm. In the top plots, the grey line corresponds to the ground reaction forces, while the graphs in blue represent pelvis vertical velocity (PVV, left) and resultant foot acceleration (RFA, right) during a sprint with deceleration task, used as an example. Initial contact (IC) and toe-off (TO) events detected by the GRF are shown with dashed lines in red. The hybrid algorithm obtained the best performance among the three.

To optimize FCD accuracy, a hybrid approach was developed based on the strengths of both algorithms (Figure 4). According to the single algorithms’ performance (Table 1), the hybrid algorithm primarily relies on RFA for detecting both IC and TO. If RFA fails to identify a valid foot contact window, PVV serves as a fallback. Additionally, when the magnitude of foot acceleration at IC is below 60 m/s^2^ (approximated from 57.8 m/s^2^, see algorithms’ performance above), the IC detected by RFA is replaced with the IC identified by PVV, while TO detection remains based on RFA.

As shown in Table 1, the hybrid algorithm achieved a median offset of −5.6 ms (IQR = 70.4 ms) for IC and 20.4 ms (IQR = 78.7 ms) for TO detection, outperforming the other two algorithms. Bland–Altman analysis (Figure 3) further confirmed the improved agreement with the reference system, showing for IC detection a reduced mean offset compared with both PVV and RFA (−8.6 ms vs. −9.8 ms for PVV and −24.7 ms for RFA) and for TO detection a reduced mean offset relative to PVV (17.6 ms vs. −105.6 ms) and comparable offset to RFA. The Linear Mixed-Effects analysis did not reveal a statistically significant model for IC (*p* = 0.205), whereas the TO model was statistically significant (R^2^ = 0.071, *p* < 0.001), with the DEC task (β = −3.785, *p* < 0.001) being associated with an increase in error (Table 2). No between-subject variability was observed.

## 4. Discussion

The present study developed and validated a novel hybrid algorithm that delivers robust and reliable IMU-based FCD during high-dynamic sports movements. The algorithm accurately identified IC and TO events, demonstrating median temporal offsets within 20 milliseconds relative to force platform measurements, the established gold standard. This level of precision confirms the algorithm’s capability to capture the critical phases of foot–ground interaction and is well within the 40–45 millisecond window reported in the literature between initial contact and the injury frame in high-dynamic tasks [22,35,36]. This indicates that the proposed algorithm is well suited for real-world field applications, offering the precision required for accurate biomechanical assessments of injury risk and performance in high-dynamic sports.

Among the initially tested approaches (Algorithms 1 and 2), the RFA algorithm was overall more accurate than PVV, especially for TO detection, but it was sensitive to low accelerations (<60 m/s^2^), resulting in decreased event detection reliability under these conditions. In contrast to previous studies reporting a significant correlation of IMU-based algorithms with speed in walking and running tasks [16,17], no such correlation was observed for the PVV algorithm. This is probably due to the high-dynamic and non-linear COD and DEC tasks analyzed in this study, which introduce greater subject variability in PVV signals, reducing their direct relationship with speed [37,38,39].

The hybrid algorithm (Algorithm 3) was designed to combine the strengths of the two previous literature-based ones, reducing detection errors and improving robustness. Minimal influence of co-factors was noted, with less than 10% explained variance in all models, suggesting that the algorithm is relatively stable across participants and conditions, despite varying anthropometric characteristics and different biomechanics. Task type, instead, significantly affected TO detection, suggesting that specific movement patterns may impact FCD performance. These findings highlight the need for task-specific algorithm adaptations to enhance generalizability across diverse movement profiles.

The developed algorithm, as implemented, potentially allows for the detection of multiple consecutive footsteps during high-dynamics movements and specifically detects the foot contact window of interest in the analyzed task, enabling reliable IMU-based biomechanical analysis for both performance and clinical field applications. The ability to accurately identify foot contact windows using wearable IMUs in real-world conditions represents a practical and accessible alternative to force platforms, especially for researchers or embedded scientists working in rehabilitation or sports settings where access to advanced laboratory equipment is limited or not feasible [6,40].

Some limitations should be acknowledged. First, the participants represented a relatively small cohort of young, healthy, and competitive athletes, which may prevent the findings from being generalized to clinical populations such as individuals with musculoskeletal impairments and altered motion patterns. Nevertheless, it is worth noting that the sample size was larger than the average reported in comparable studies using IMU-based methodologies [41]. Second, the thresholds and detection rules for both the PVV and RFA algorithms were tailored to the specific characteristics of the two movement tasks. While this approach allowed task-specific optimization, it may limit the applicability of the algorithm to other high-dynamic movements with different signal patterns. However, cutting movements and sprints, such as the COD and DEC tasks selected for this study, cover a conspicuous portion of high-dynamics movement tested on the field, e.g., in the late phase rehabilitation in athletes who underwent ACL reconstruction. These movements are associated with high mechanical stress on the knee and an increased risk of injury or re-injury [20]. Therefore, the proposed algorithm for FCD has the potential to support objective and ecological assessments of movement quality and readiness for return-to-sport, offering valuable insights for clinicians and practitioners involved in rehabilitation and performance monitoring.

## 5. Conclusions

An IMU-based algorithm integrating pelvis vertical velocity and resultant foot acceleration signals was developed and rigorously validated for foot contact detection during high-dynamic sports movements. The algorithm demonstrates strong reliability and offers a robust alternative to force platforms, enabling accurate detection of foot contact events and the analysis of multidirectional movements in ecological sports environments.

## Figures and Tables

**Table 1:** Statistical analysis results are reported as median error (force data—IMU data) and interquartile range (IQR), also expressed as [Q1; Q3], for both IC and TO detection using the PVV algorithm, the RFA algorithm, and the final hybrid algorithm. Results are expressed in milliseconds (ms). Positive values indicate that the algorithm detected the foot contact event earlier than the reference system.

	Median Error [ms]	IQR [ms]
**IC**		
PVV	−16.7	77.8 [−57.4; 20.4]
RFA	−10.2	76.8 [−50.9; 25.9]
Hybrid	−5.6	70.4 [−42.6; 27.8]
**TO**		
PVV	102.8	84.3 [70.4; 154.6]
RFA	20.4	78.7 [−18.5; 60.2]
Hybrid	20.4	78.7 [−18.5; 60.2]

**Table 2:** Results of multivariate linear regression model for the three algorithms (PVV, RFA, and hybrid) for both IC and TO. Note: COD = 90° change of direction.

	PVV	RFA	Hybrid
**IC**	**R^2^ = −0.008, *p* = 0.822**	**R^2^ = 0.072, *p* < 0.001**	**R^2^ = 0.006, *p* = 0.205**
Task (COD)	β = −0.408, *p* = 0.791	β = 0.243, *p* = 0.737	β = 0.092, *p* = 0.913
Sex (Male)	β = −0.064, *p* = 0.966	**β = 3.242, *p* < 0.001**	β = 1.682, *p* = 0.045
Leg (Non-dominant)	β = −1.405, *p* = 0.357	β = 0.840, *p* = 0.243	β = 0.524, *p* = 0.532
**TO**	**R^2^ = −0.002, *p* = 0.465**	**R^2^ = 0.071, *p* < 0.001**	**R^2^ = 0.071, *p* < 0.001**
Task (COD)	β = −1.276, *p* = 0.392	**β = −3.785, *p* < 0.001**	**β = −3.785** **, *p* < 0.001**
Sex (Male)	β = −0.638, *p* = 0.666	β = 1.034, *p* = 0.210	β = 1.034, *p* = 0.210
Leg (Non-dominant)	β = −1.858, *p* = 0.210	β = 0.394, *p* = 0.633	β = 0.394, *p* = 0.633

**Figure A1:**
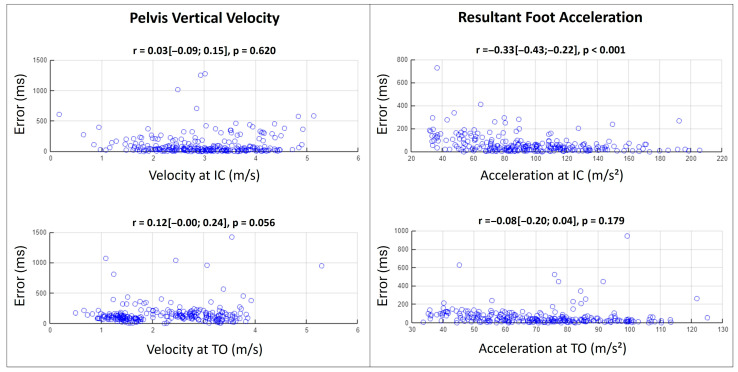
Correlation analysis between velocity at IC and TO with the PVV algorithm’s error (**left**) and between foot acceleration magnitude and the RFA algorithm’s error (**right**). Pearson correlation coefficients (r), 95% confidence intervals, and *p*-values are reported for each analysis.

## Data Availability

The original contributions presented in this study are included in the article. Further inquiries can be directed to the corresponding author.

## Abbreviations

The following abbreviations are used in this manuscript:

FCD	Foot Contact Detection
IMU	Inertial Measurement Unit
ACL	Anterior Cruciate Ligament
IC	Initial Contact
TO	Toe-Off
vGRF	Vertical Ground Reaction Force
PVV	Pelvis Vertical Velocity
RFA	Resultant Foot Acceleration
COD	90° Change of Direction
DEC	Linear Sprint with Deceleration
IQR	Interquartile Range
